# Brief intervention for alcohol misuse in people attending sexual health clinics: study protocol for a randomized controlled trial

**DOI:** 10.1186/1745-6215-13-149

**Published:** 2012-08-25

**Authors:** Rahil Sanatinia, Barbara Barrett, Sarah Byford, Madeleine Dean, John Green, Rachel Jones, Baptiste Leurent, Anne Lingford-Hughes, Michael Sweeting, Robin Touquet, Peter Tyrer, Helen Ward, Mike J Crawford

**Affiliations:** 1 Centre for Mental Health, Imperial College, London, UK; 2 Centre for the Economics of Mental and Physical Health, King’s College, London, UK; 3 Central and North West London NHS Foundation Trust, London, UK; 4 Chelsea and Westminster hospital NHS Foundation Trust, London, UK; 5 PRIMENT Clinical Trials Unit, University College, London, UK; 6 Neuropsychopharmacology Unit, Imperial College, London, UK; 7 MRC Biostatistics Unit, Cambridge, UK; 8 Imperial College Healthcare NHS Foundation Trust, London, UK; 9 School of Public Health, Imperial College, London, UK

**Keywords:** Alcohol misuse, Intervention, Randomized controlled trial, Sexual health, Effectiveness

## Abstract

**Background:**

Over the last 30 years the number of people who drink alcohol at harmful levels has increased in many countries. There have also been large increases in rates of sexually transmitted infections. Available evidence suggests that excessive alcohol consumption and poor sexual health may be linked. The prevalence of harmful alcohol use is higher among people attending sexual health clinics than in the general population, and a third of those attending clinics state that alcohol use affects whether they have unprotected sex. Previous research has demonstrated that brief intervention for alcohol misuse in other medical settings can lead to behavioral change, but the clinical- and cost-effectiveness of this intervention on sexual behavior have not been examined.

**Methods:**

We will conduct a two parallel-arm, randomized trial. A consecutive sample of people attending three sexual health clinics in London and willing to participate in the study will be screened for excessive alcohol consumption. Participants identified as drinking excessively will then be allocated to either active treatment (Brief Advice and referral for Brief Intervention) or control treatment (a leaflet on healthy living). Randomization will be via an independent and remote telephone randomization service and will be stratified by study clinic. Brief Advice will comprise feedback on the possible health consequences of excessive alcohol consumption, written information about alcohol and the offer of an appointment for further assessment and Brief Intervention. Follow-up data on alcohol use, sexual behavior, health related quality of life and service use will be collected by a researcher masked to allocation status six months later. The primary outcome for the study is mean weekly alcohol consumption during the previous three months, and the main secondary outcome is the proportion of participants who report unprotected sex during this period.

**Discussion:**

Opportunistic intervention for excessive alcohol use has been shown to be effective in a range of medical settings. The SHEAR study will examine whether delivering such interventions in sexual health clinics results in reductions in alcohol consumption and will explore whether this is associated with changes in sexual behavior.

## Background

Concerns have been raised regarding increased levels of alcohol consumption in many countries [1]. It is estimated that as many as one in five adults drink too much alcohol in the UK [2]. This may take the form of sustained excessive consumption or episodic bouts of ‘binge’ drinking. Excessive alcohol consumption can lead to a range of physical and mental health problems, which result in increased use of healthcare services and costs to society associated with sickness absence, unemployment, accidents and crime [3].

The proportion of people who drink excessively has increased considerably over the last 30 years, especially among people aged under 25 [4]. Increasing levels of alcohol misuse have been accompanied by large increases in rates of sexually transmitted infections [5]. The figures published by the Health Protection Agency (HPA) in 2010 showed a record level of nearly half a million new diagnoses. While we do not know if alcohol use is implicated in this increase, epidemiological data suggest that it may be. For instance, cross-sectional data from a sample of over 1,000 young people in nine European cities demonstrated that alcohol consumption was associated with the number of sexual partners and age at first sexual contact [6]. In the United States, changes in the price of alcohol in the 1980s and 1990s were highly correlated with changes in rates of gonorrhoea [7]. Policies which succeeded in reducing drunk driving rates in young men in the United States were associated with subsequent reductions in gonorrhoea rates among young males [8]. Further evidence to support an association between alcohol consumption and sexual behavior comes from cross-sectional surveys, which have also demonstrated high levels of alcohol consumption among people attending sexual health clinics [9-11]. In one study, over a third of participants indicated that their attendance was alcohol related [10].

Systematic reviews of brief interventions for alcohol misuse have shown that they lead to clinically important reductions in alcohol consumption across a range of healthcare settings [12,13]. Interventions delivered in a single session appear to be as effective as more lengthy ones [14]. Stepped interventions in which people receive interventions of greater intensity depending on the extent of their needs have also demonstrated positive effects [15,16].

To date, there has been only one randomized trial of brief intervention for alcohol misuse in a sexual health setting [17]. In this study, half of a total sample of 184 people found to be drinking excessively was offered a brief intervention delivered by one of two trained nurses. At a three-month follow-up 62 % of those that received the brief intervention reported consuming less alcohol compared to 47 % of those in the control arm of the trial. While this study demonstrated the feasibility of a randomized trial of brief alcohol intervention for people attending sexual health clinics, the intervention was delivered by a research nurse, rather than those already working in the clinic, limiting the generalizability of the study findings. In addition, the impact of brief intervention on sexual health outcomes was not explored and the costs and cost-effectiveness of the intervention were not examined. There is widespread recognition of the need for experimental studies to examine the clinical effects and cost-effectiveness of screening and brief intervention for alcohol misuse for people who attend sexual health clinics [18].

### Research objectives

The aim of the SHEAR study (Sexual Health and Excessive Alcohol: Randomized trial) is to examine the effectiveness and cost-effectiveness of opportunistic brief intervention for alcohol misuse for people who attend sexual health clinics and consume excessive alcohol. To achieve this aim we will:

1. Test whether brief intervention reduces subsequent alcohol consumption measured six months later compared to the control treatment.

2. Examine whether brief intervention compared to control treatment is associated with changes in sexual behavior.

3. Examine the cost-effectiveness of brief intervention compared to control treatment.

#### Hypotheses

i. Brief intervention for those attending sexual health clinics and drinking excessively reduces mean weekly alcohol consumption over a 12-week period prior to the 6-month follow-up interview (that is, weeks 13 to 24 after intervention).

ii. Brief intervention for those attending sexual health clinics and drinking excessively reduces the likelihood of unprotected sexual intercourse over a 12-week period prior to the 6-month follow-up interview.

iii. Brief intervention for those attending sexual health clinics and drinking excessively is more cost-effective than control treatment.

## Methods/Design

The design is a two parallel-arm, single-blind, individually randomized controlled trial. The trial will be an integrated clinical and economic evaluation and will compare the intervention versus control treatment on alcohol consumption, sexual behavior, health-related quality of life and costs in the six months following randomization. We will also conduct a parallel process evaluation, which will include an examination of the uptake of study interventions and an analysis of qualitative data from in-depth interviews with a sample of service users and providers. These data will help us to examine the uptake and acceptability of the interventions being used and explore the relationship between the study context and outcomes.

### Study setting

Study participants will be recruited from three sexual health clinics in London. Collectively, these clinics serve a diverse population with high levels of alcohol misuse and poor sexual health [19,20].

### Recruitment

**Figure 1:**
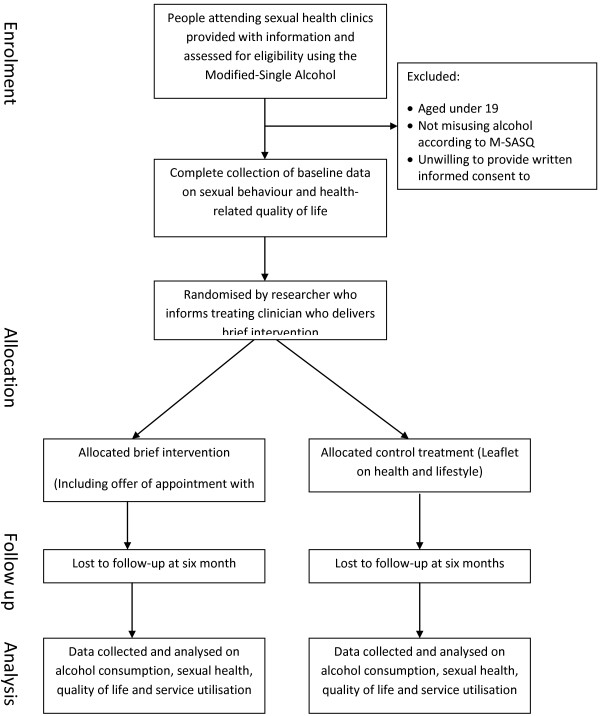
Recruitment flow diagram.

At each clinic where we recruit participants, information about the study will be displayed on posters in waiting rooms. On days when recruitment is taking place, clinic staff will hand all those attending the service a postcard with information about the study and ask people if they would be willing to meet a researcher. If they agree to this, a researcher will explain the rationale for the study and give them a copy of the Patient Information Leaflet. The Patient Information Leaflet states that we are conducting a study about ‘sexual health and lifestyle advice’ and only refers to alcohol in the context of other aspects of lifestyle, such as exercise, diet and smoking. The researcher will encourage potential participants to spend as much time as they want asking questions about the study and considering whether they wish to take part or not. Before any trial specific procedures are performed, the patient will sign and date the Informed Consent Form. For those willing to provide consent, eligibility to participate in the study will be assessed and baseline clinical and demographic data will be collected. Potential participants will be asked five questions on ‘health and lifestyle’. We will check with participants the contact details they have given to clinic staff to make sure they are correct. We will ask participants for other information that may assist follow-up and seek written informed consent to contact their general practitioner solely for the purpose of helping us collect follow-up data. A recruitment flow chart is presented in Figure 1.

### Randomization

Those who are eligible will be randomized to brief intervention or control treatment. The researcher will provide those who are ineligible with written information about health and lifestyle. Remote telephone randomization will be undertaken through a fully automated telephone-based service operated by an independent Clinical Trials Unit. Equal numbers of participants will be randomized to each arm of the trial. We will use permuted blocks stratified by the clinic, with block size randomly assigned to between four and six. The researcher will notify the treating clinician concerning which arm of the trial they are in. This researcher will play no part in the collection of follow-up data.

### Follow-up

Three months after randomization, study participants will receive a phone call, text message or an email thanking them for taking part in the study, reminding them that they will be contacted in three months’ time to complete the follow-up interview, and asking them to let us know whether their contact details are likely to change during this period. If, at six months, our attempts to contact a participant are unsuccessful, we will check their contact details against those given during any subsequent visits to the clinic. If the participant provided consent for us to contact their general practitioner, a family member or friend we will contact them to obtain updated contact details for the participant. Follow-up interviews will be carried out by telephone, unless the participant requests a face-to-face interview. Any face-to-face interview will take place at the clinic at a time which is convenient for the participant. Any travel costs or other reasonable expenses incurred by the participant will be reimbursed. All participants will be offered a £15 honoraria after completion of the six-month follow-up interview.

### Planned interventions

We will test the effects of Brief Advice and referral delivered by the treating clinician. The intervention is based on that used in a previous trial conducted in an emergency medical setting [15]. The intervention consists of:

· Confirming the current level of alcohol use and brief feedback that alcohol use at that level has the potential to harm health

· Making a link between alcohol and clinic attendance

· Written information on alcohol and health in the form of a leaflet recommended by the Department of Health: *‘How much is too much’*[21]

· The offer of an appointment with an Alcohol Health Worker (AHW).

This form of intervention was previously tested in a feasibility study in a sexual health clinic and found to be acceptable to clinicians working in this busy clinical setting [9].

On days when participants are recruited from the clinics, an Alcohol Health Worker will be available to see those who are willing to receive further help. Brief Intervention delivered by the AHW will last up to 30 minutes and use the ‘FRAMES’ approach [14,22]. For any participant who is drinking at a harmful or dependent level, the AHW will have the option of arranging a follow-up appointment or referring them on to local alcohol services for individual alcohol counseling, detoxification and so on. In the event that the participant is unable to attend an appointment that day they will be offered an appointment on a later date or telephone-based support and advice.

#### Control treatment

Those randomized to control treatment will be offered a copy of the leaflet *‘Five Choices to Help You Stay Healthy’*[23], which provides general information on preventative health, including alcohol use, diet, exercise, cigarette smoking and details of how to obtain further information about health and lifestyle.

### Treatment integrity

Treatment integrity will be maximized through training, supervision and checks on written notes kept by clinicians and Alcohol Health Workers. All clinicians who deliver the brief intervention will receive training in accordance with Department of Health guidelines (http://www.alcohollearningcentre.org.uk). All Alcohol Health Workers who take part in the study will be experienced practitioners who have undertaken specific training in counseling people who misuse alcohol. All Alcohol Health Workers will receive regular clinical supervision. Clinical supervisors will encourage Alcohol Health Workers to discuss work with trial participants along with other patients they see.

Front-line clinicians will be asked to complete a short tick box proforma for each study participant to indicate whether the four components of the Brief Advice and referral are delivered. Alcohol Health Workers will be asked to complete a longer proforma which will record the number and length of session(s), interventions delivered during the session(s) and further information on referrals that were subsequently made. The proforma is based on that used to assess treatment fidelity in our previous trial of brief intervention in the Accident and Emergency Department [15]. At the end of the study, we will inspect a random sample of 20% of all study proforma to identify the proportion of people in the experimental arm of the trial who receive the interventions they were allocated.

### Planned inclusion/exclusion criteria

To maximize generalizability of study findings, we have kept our inclusion criteria broad and limited our exclusion criteria. To participate in the study people must be aged 19 years or above, be drinking excessively according to the Modified-Single Alcohol Screening Question [24] and be willing to provide written informed consent to take part in the study. The age limit is because people younger than 19 years attending sexual health clinics receive health advice. We will exclude any person who is unable to communicate in English sufficiently to complete baseline questionnaires, anyone who does not have an address or contact telephone number, and anyone who believes they may not be contactable in six months’ time.

### Measures

#### Baseline measures

Basic demographic data on age, gender, and ethnicity will be extracted from clinic records and checked with the patients to make sure they are correct.

All patients consenting to take part will be asked to complete a computer-assisted self-completion interview [25,26].

Alcohol consumption will be assessed using the Modified-Single Alcohol Screening Question (M-SASQ). The M-SASQ is a brief validated measure of harmful alcohol use that is acceptable to patients in general medical settings [24]. It consists of a single question - for men: ‘How often do you drink more than eight units of alcohol on one occasion?’ and for women: ‘How often do you drink more than six units of alcohol on one occasion?’ To help people answer this question they are shown a card which describes what a unit of alcohol is. Those drinking at such levels once a month or more are considered eligible. The question on alcohol is embedded in a series of four other questions asking about diet, exercise and smoking.

In addition, eligible patients will be prompted to answer questions on their sexual behavior and health-related quality of life (EuroQol 5 Dimensions (EQ-5D) [27]).

### Follow-up measures

Follow-up data will be obtained by a telephone interview by a researcher who is masked to the participant’s allocation status.

Alcohol consumption data in the last 90 days will be collected using the Form 90. The Form 90 is a validated alcohol consumption assessment tool which provides a detailed day-by-day account of alcohol use in the 90 days prior to the interview [28].

In addition, participants will be asked about hazardous drinking (Alcohol Use Disorders Identification Test-Consumption (AUDIT-C) [29]), sexual behavior in the last three months, health-related quality of life (EQ-5D [27]) and resource use during the previous six months (Adult Service Use Schedule [30]).

### Outcomes

1. Primary: Mean weekly units of alcohol consumed during the previous 90 days using the Form 90 [28].

2. Main secondary: Whether or not the participant has had unprotected sex in the last three months.

3. Other secondary: Mean units consumed per drinking day and percentage days abstinent, both measured using the Form 90, and whether or not the participant is drinking alcohol at hazardous levels using the AUDIT-C [29].

Our secondary sexual behavior outcomes are based on those used in a previously reported study [31] and will comprise: total number of sexual partners during the last three months, any incidence of regretted sex in the last three months, number of people they had unprotected sex with (anal or vaginal sex without a condom) in the three months before the interview, any incidence of unprotected sex while drunk in the last three months, how long they knew their last sexual partner before they had sex with them, unwanted pregnancy and any new diagnosis of a sexually transmitted infection in the last three months.

### Sample size

Initial sample size calculations were based on comparison of the primary outcome. In order to achieve 80% power to detect a mean weekly difference of 23.4 units of alcohol consumption with standard deviation of 58 units [15], 97 evaluable participants per arm were needed. An inflation factor of 1.15 for clustering by clinician in the intervention arm was considered [32], based on an intracluster correlation coefficient of 0.04 [33] and a cluster size of 7 in the intervention arm. Together with an anticipated 30 % drop-out rate at six months, the initial recruitment target was, therefore, 160 per arm.The first months of the trial showed that recruitment and retention rates were higher than expected; the sample size was, therefore, modified to provide additional power to test both the primary and main secondary hypotheses (a reduction in unprotected sexual intercourse) of interest.

The final sample size was based on a practical size of 380 per arm (760 in total). If 65% of participants had unprotected sex in the control group compared to 50% in the intervention arm, the power to detect such an effect would be above 90%, assuming a 25% drop out, and a clustering design effect of 1.15. The power would remain above 80% if the absolute difference is 13%.

### Statistical analysis

Baseline characteristics will be reported by treatment arm. The primary outcome will be analyzed using random-effects linear regression adjusted for age, sex and harmful alcohol use measured at baseline, testing for the presence of an intervention effect between the two arms. The random-effects model will take into account clustering by sexual health clinic and, in the intervention arm, by treating clinician [34].

Alcohol consumption is not expected to be normally distributed and particular care will be taken to check the validity of the regression, including normality and homoscedasticity of the residuals. If the assumptions are violated, sensitivity analysis will be performed as appropriate.

Participants who dropped out from the trial will be compared to the completers. Sensitivity of the results to missing data will be assessed by performing multiple imputations; further models allowing for missing not at random mechanism will also be considered [35].

The main secondary outcome will be compared in the same way as the primary, using logistic regression and adjusting for unprotected sex at baseline. Other secondary outcomes will be compared using linear or logistic regression, and adjusted for baseline value, as appropriate.

In order to assess for possible heterogeneity of the intervention effect, primary and main secondary outcomes will also be reported by the following subgroups: gender, age (<25, 25 to 35, 35 and older), ethnicity (white vs. other), sexual orientation (heterosexual vs. other). All analyses will be performed according to the randomization arm (intention-to-treat), and two-sided *P*-values considered significant when below 0.05.

The economic evaluation will take the NHS/Personal Social Services perspective preferred by the National Institute for Health and Clinical Excellence [36] and shown to be the key cost sectors in our previous research of brief intervention for alcohol misuse [30]. Data on uptake of the brief intervention will be collected from records to avoid patients revealing their treatment group to the research assessors. Data on indirect time, including preparation and supervision, will be collected directly from the treating clinician. Data on the use of other health and social services will be collected using the Adult Service Use Schedule (AD-SUS) adapted for alcohol misuse in previous research [30]. The cost of the brief intervention will be directly calculated from salaries using a micro-costing approach [37]. National UK unit costs will be applied to medication, hospital contacts and community health and social services [38,39].

Differences in mean costs will be analyzed using standard parametric t-tests with the validity of results confirmed using bias-corrected, nonparametric bootstrapping (repeat re-sampling) [40]. Despite the skewed nature of cost data, this approach is recommended to enable inferences to be made about the arithmetic mean [41]. Cost-effectiveness will be assessed through the calculation of incremental cost-effectiveness ratios [42] and will be explored in terms of alcohol consumption (primary economic analysis) and quality adjusted life years (QALYs) using the EQ-5D measure of health-related quality of life (secondary economic analysis). Uncertainty around the cost and effectiveness estimates will be represented by cost-effectiveness acceptability curves [43].

### Ethical issues

Approval for the study was obtained from West London Research Ethics Committee 3 (10/H0706/29) and The Research and Development departments of Chelsea and Westminster Hospital NHS Foundation Trust and Imperial College Healthcare NHS Trust prior to the start of data collection at each local clinic. Only those who agree to provide written informed consent will be included in the study. Each potential participant will be provided with a copy of an information sheet that includes a contact number for the study team.

## Discussion

The SHEAR study is the first large-scale randomized trial to examine the clinical and cost-effectiveness of brief intervention for alcohol misuse for people attending sexual health clinics. We are testing a very brief approach to screening and intervention that is aimed at accurately identifying people who are drinking excessively and helping them reduce their drinking while minimizing any disruption to normal clinical practice. The use of a short (single-item) screening question may have other benefits. It has been argued that previous trials of brief intervention for excessive alcohol use may underestimate the impact of ‘active’ treatment because the process of assessing alcohol use may itself highlight this issue and prompt people to reflect on and consider reducing the amount they drink [44]. In the SHEAR study all material used to publicize and recruit participants refers to alcohol in the context of other lifestyle factors that affect health, such as smoking and diet. We believe that this approach better represents the absence of enquiry or information about alcohol that people currently receive in this and other medical settings, thus allowing us to examine the full impact associated with the brief intervention we are testing.

The form of Brief Advice and referral that we are using is offered to participants after their main health concerns have already been met [45] and includes a brief statement about health consequences of alcohol use, an approach which has been demonstrated to increase uptake of brief alcohol interventions [46]. While this form of intervention has been shown to be acceptable to patients in other settings, we know very little about acceptability in sexual health clinics. We will use quantitative data on levels of uptake of interventions and qualitative data from in-depth interviews with service users and providers to examine the acceptability of screening and Brief Advice to people who are found to be drinking excessively in this setting.

In addition to being large enough to demonstrate reductions in alcohol consumption of a magnitude seen in previous trials, the relatively large sample size means that we have sufficient power to examine clinically important changes in sexual behavior associated with this intervention. While reducing the level of alcohol misuse is worthwhile, the widespread uptake of brief intervention across sexual health clinics may require evidence that any change in drinking are associated with changes in sexual behavior [18]. However, we are only collecting data from people attending three sexual health clinics in London. The findings might not be generalizable to those in rural areas or people with different cultural and socioeconomic backgrounds.

The study faces a number of important challenges, including the need to ensure the fidelity of a brief intervention delivered by over 30 clinicians working across three different clinics, and the need to achieve a high follow-up rate in a relatively young and mobile study population. If we are able to meet these challenges, we will generate data about the clinical- and cost-effectiveness of a form of brief screening and intervention that has the potential to be delivered more widely across sexual health clinics and help establish whether the previously reported association between high alcohol consumption and poor sexual health is a causal one.

### Trial status

Recruitment is on-going (800 participants recruited as of end of April 2012).

## Abbreviations

AD-SUS: Adult Service Use Schedule; AHW: Alcohol Health Worker; AUDIT-C: Alcohol Use Disorders Identification Test-Consumption; EQ-5D: EuroQol 5 Dimensions; M-SASQ: Modified-Single Alcohol Screening Question.

## Competing interests

The authors declare that they have no competing interests.

## Authors’ contributions

MC,, RS, BB, SB, JG, LB, MS, RT and HW contributed to the design of the study. All authors contributed to development of the study protocol. MC and RS drafted the manuscript. All authors provided a critical review and final approval of the manuscript.
